# An Atypical Anomalous Aortic Origin of the Left Coronary Artery With Intra-Arterial Wall Course Pretending a Normal Migration on Imaging Screening: A Case Report

**DOI:** 10.3389/fcvm.2022.918832

**Published:** 2022-06-09

**Authors:** Fan Hu, Xinyue Wang, Jiaojiao Wan, Yifei Li, Tao Wang, Kaiyu Zhou, Xiaoqing Shi, Zhongqiang Liu, Jie Fang, Yimin Hua

**Affiliations:** Key Laboratory of Birth Defects and Related Diseases of Women and Children of MOE, State Key Laboratory of Oral Diseases, Department of Pediatrics, National Clinical Research Center for Oral Diseases, West China Second University Hospital, West China Hospital of Stomatology, Sichuan University, Chengdu, China

**Keywords:** anomalous aortic origin of a coronary artery, non-coronary sinus of valsalva, intra-arterial wall course, syncope, case report

## Abstract

**Background:**

An anomalous aortic origin of a coronary artery (AAOCA) has been considered as a dominant cause of sudden cardiac death (SCD) among young age children. Therefore, it is critical to identify AAOCA timely to avoid lethal events. Recently, accumulating cases of right or left coronary arteries originating from inappropriate locations at the sinus of Valsalva have been identified. Here, we report a rare case of AAOCA with an intra-arterial wall course pretending normal migration on imaging screening in a patient who suffered from syncope.

**Case summary:**

A 7-year-old male without a previous history of cardiovascular and cerebrovascular diseases suddenly suffered from sharp chest pain and syncope after intensive exercise. The electrocardiogram showed that the ST segment of multiple leads was depressed by more than 0.05 mV, and biomarkers indicated severe myocardial injuries. The left ventricular ejection fraction (LVEF) decreased dramatically to 23%. Fulminant myocarditis and cardiomyopathy were therefore excluded. However, a relatively normal coronary artery origin, which arose from the left coronary sinus, presented on echocardiography and cardiac CT angiography (CTA). It is difficult to draw an association between severe clinical manifestations and slight malformations on echocardiography and CTA. Furthermore, selective coronary angiography revealed that an anomalous left coronary artery arose from the superior margin of the inappropriate sinus, developed an intramural wall course and finally exits the left sinus of Valsalva and migrated between the aorta and the pulmonary artery, which induced severe myocardial infarction during exercise. Then, the patient received surgical correction with a modified unroofing procedure. After 2 months of intensive treatment, the patient was discharged and remained asymptomatic through 18 months of follow-up.

**Conclusion:**

AAOCA, especially anomalous left coronary artery (ALCA), represents a major potential risk of SCD. We reported an atypical manifestation of ALCA arising from the inappropriate sinus of Valsalva and merging into the intra-arterial wall to develop a strange course and then sprout between the aorta and the pulmonary artery. The diversity of AAOCA might present as a relatively normal course under non-invasive radiological imaging scanning.

## Introduction

Congenital anatomical variation of the coronary artery can be classified as abnormalities of coronary artery origin, course, destination, and various vessels (1). Due to the complex phenotypes of congenital coronary artery malformation, various clinical manifestations can be observed. Some types of malformations can be recognized as benign variations, as coronary artery perfusion and circulation maintain physiological function. Adverse outcomes occur at a low incidence. However, in some cases of coronary artery malformation lead to severe myocardial ischemia and result in recurrent syncope and even sudden cardiac death (SCD) (2, 3). Moreover, anomalous aortic origin of a coronary artery (AAOCA) has been considered as a dominant cause of SCD at a young age (4–6). Therefore, it is critical to identify AAOCA timely to avoid lethal events.

Recently, several cases of right or left coronary arteries originating from inappropriate locations at the sinus of Valsalva have been reported. Generally, the majority of patients with anomalous left coronary artery (ALCA), which originates from the sinus of Valsalva, demonstrated poor prognosis, especially for those patients who suffered from an interarterial segment coursing between the aorta and the pulmonary artery, predicting a relatively high incidence of syncope, myocardial infarction and SCD (7, 8). With the application of CT angiography (CTA) and cardiac MRI scanning, a greater proportion of ALCA can be identified with non-invasive protocols (9). The guidelines for adult treatment of congenital heart disease issued by the American Cardiology Association (ACC) and the American Heart Association (AHA) point out that the treatment for all patients with left coronary arteries originating from the inappropriate coronary sinus and abnormal course between the aorta and the pulmonary artery should be surgical revascularization as early as possible (10). Unfortunately, AAOCA is often misdiagnosed as fulminant myocarditis or cardiomyopathy. Therefore, the rapid and accurate diagnosis of AAOCA is associated with advanced prognosis for such patients to avoid SCD.

Here, we report a rare case of AAOCA with an intramural course pretending normal migration on imaging screening who suffered syncope and SCD. This patient presented a relatively normal coronary artery migration on echocardiography. Furthermore, CTA demonstrated significant dysplasia of the left coronary artery, but it remained in the typical course. Besides, electrocardiogram (EKG) and cardiac MRI revealed severe myocardial ischemia. Finally, transcatheter angiography for the coronary artery was performed to identify an intramural course of left coronary artery and exits the left sinus of Valsalva, giving the impression of normal origin by echocardiography.

## Case Presentation

### Ethical Compliance

This report was approved by the Ethics Committee of the West China Second Hospital of Sichuan University (approval number 2014-034). Informed consent was obtained from the patient’s parents prior to performing whole exon sequencing and for the inclusion of the patient’s clinical and imaging details in subsequent publications.

### History of Illness

A 7-year-old boy suddenly suffered sharp chest pain and syncope during athletic training of fast-running for 5 min. Timely cardiopulmonary resuscitation (CPR) was performed by the coach before the arrival of ambulance. The patient was transferred to our cardiac intensive care unit within 30 min. The patient denied any history of past cardiovascular and cerebrovascular illness. His parents also had no positive and related family history of arrhythmia, cardiomyopathy, congenital heart disease or coronary artery diseases. However, when systematically reviewing his past illness history, we found several transient manifestations of dizziness and amaurosis during exercise in the most recent year.

### Physical, Laboratory, Imaging Examination, and Surgical Treatment

**FIGURE 1:**
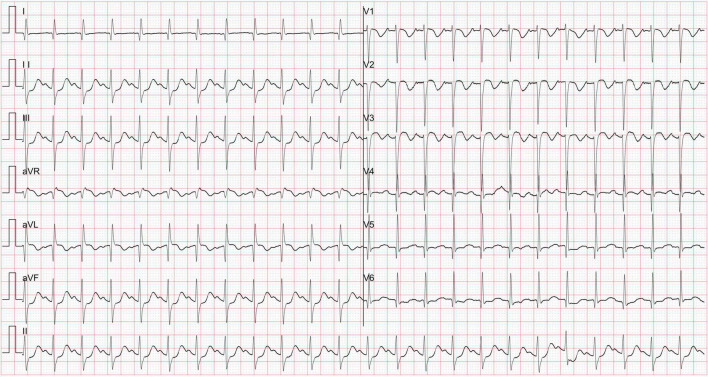
The electrocardiogram global ST segment depression of leads II, III, aVF and V3-V6 was more than 0.05 mV, and abnormal Q waves were observed.

**FIGURE 2:**
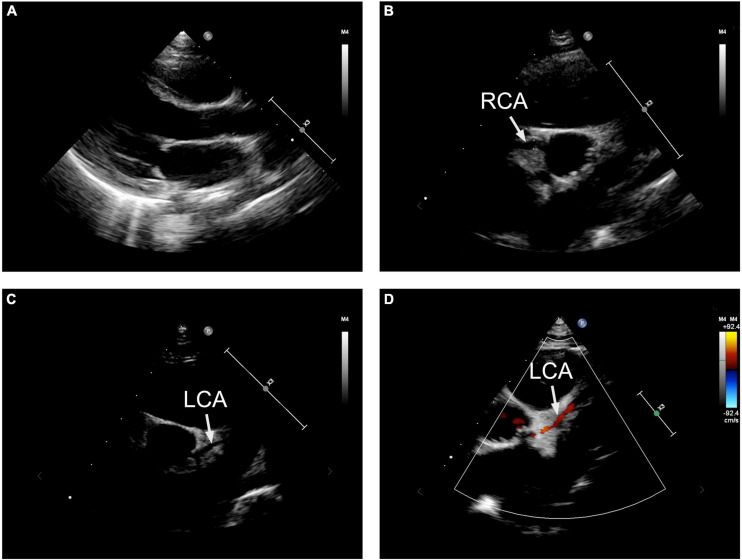
The echocardiographic presentation. **(A)** Left ventricular ejection fraction (LVEF) decreased dramatically to 23%. **(B)** The right coronary artery presented clearly with normal formation and lumen diameter. **(C)** The origin and course of the left coronary artery seemed to be normal, with a related narrowed lumen diameter. **(D)** Doppler demonstrated a non-continuous blood flow in the left coronary artery. RCA, right coronary artery; LCA, left coronary artery.

The patient presented with severe cardiac dysfunction with NYHA class IV heart function. The initial EKG measurement at hospital administration showed global ST segment depression of leads II, III, aVF, and V3-V6 of more than 0.05 mV and abnormal Q waves (Figure 1), which revealed myocardial ischemia. In addition, the serum level of cardiac troponin I (cTnI) was greater than the upper threshold (>50.00 μg/L, n.v. <0.06 μg/L) and significantly elevated B-type natriuretic peptide (BNP) at 1157.14 pg/mL (n.v. <100 pg/mL). Fulminant myocarditis was ruled out based on negative results for all tests for suspected virus and EKG changes. Echocardiography demonstrated a normal cardiac structure with a mildly enlarged left ventricle. However, his left ventricular ejection fraction (LVEF) decreased dramatically to 23% (Figure 2A). Unfortunately, there was no evidence of myocardial hypertrophy, pathological ventricular dilatation, or restriction of cardiac diastolic movements. Therefore, there was no convincing evidence for a diagnosis of cardiomyopathy.

**FIGURE 3:**
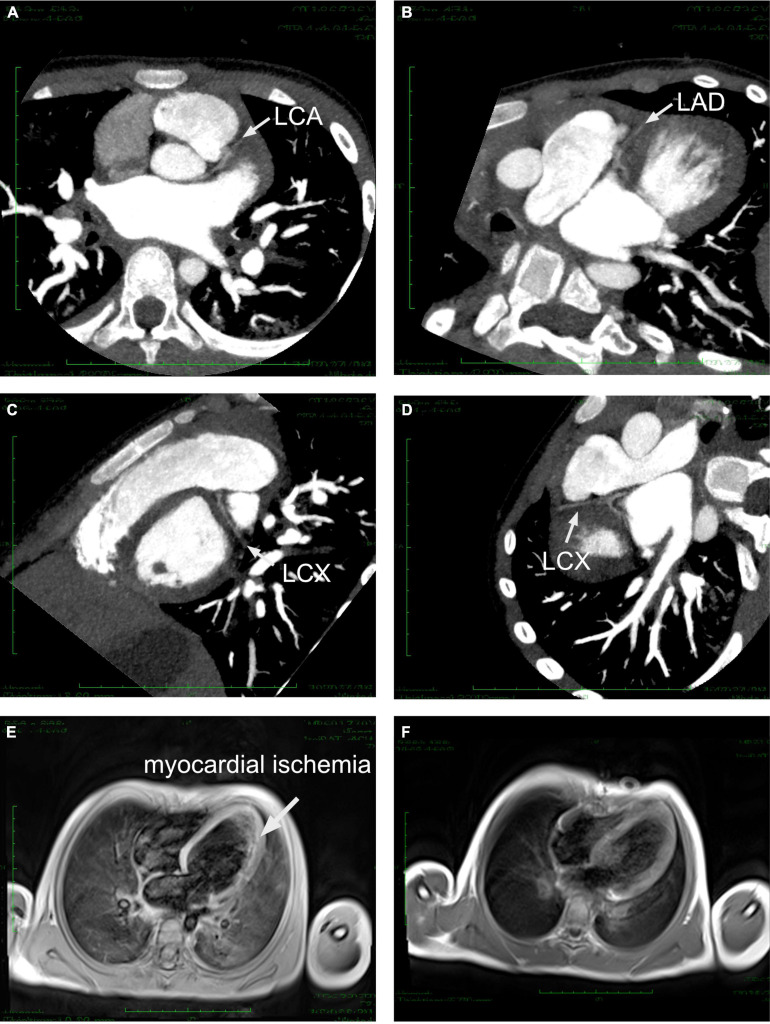
Cardiac CTA and MRI imaging. **(A)** Left coronary artery originated from an abnormal location at the superior and posterior sites in the left coronary sinus. **(B–D)** LAD and LCX demonstrated severe dysplasia under several sections of CTA. **(E)** Cardiac MRI demonstrated significant myocardial ischemia and fibrosis in the left ventricular wall before surgical correction. **(F)** Cardiac MRI image after surgical correction revealed a normal perfusion. LCA, left coronary artery; LAD, left anterior descending; LCX, left circumflex.

According to the diagnostic flow chart for SCD and myocardial ischemia, any malformation of the coronary artery was suspected. Therefore, echocardiography was used to scan the origins of the right and left coronary arteries. In these images, the right coronary artery clearly presented with normal formation and lumen diameter (Figure 2B). The origin and course of the left coronary artery seemed to be normal, presenting a slightly narrowed lumen diameter (Figure 2C). However, Doppler demonstrated non-continuous blood flow in the left coronary artery (Figure 2D), which indicated dysfunction of the left coronary artery. Vasculitis or dysplasia of coronary arteries was carefully reviewed. Laboratory tests demonstrated normal levels of C-reactive protein, ESR, anti-O-streptolysin, cytokines (including IL2, IL-6, IL-12, and TNF-α) and autoimmune antibodies, which excluded a diagnosis of vasculitis. CTA was performed to determine the overall shape of the left coronary artery. The results of CTA from diverse sections of the heart revealed that the left coronary artery originated from an abnormal location at the superior and posterior sites in the left coronary sinus (Figure 3A). Importantly, the CTA images presented dysplasia of the left coronary artery (Figures 3B–D). So that, ALCA-induced coronary artery dysplasia had been suspected. Furthermore, cardiac MRI demonstrated significant myocardial ischemia and fibrosis in the left ventricular wall (Figure 3E).

**FIGURE 4:**
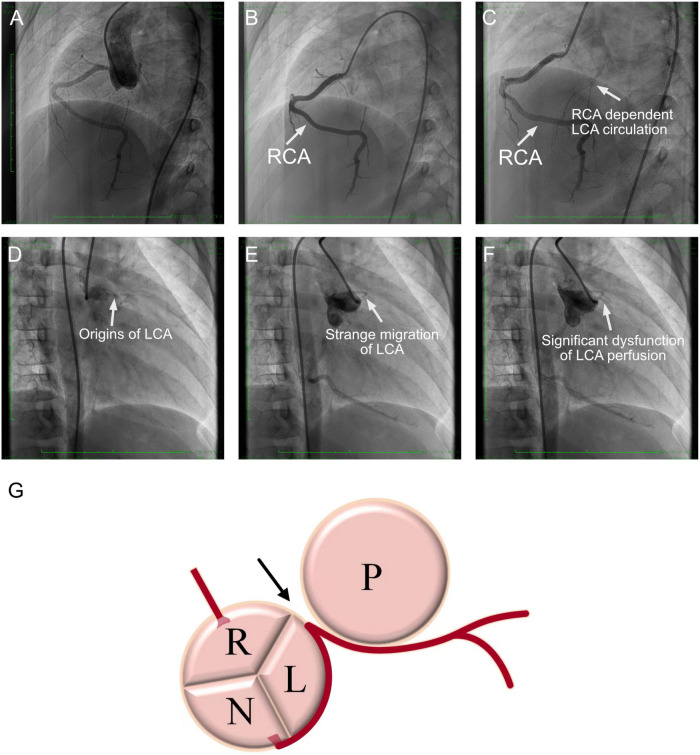
Coronary angiography images. **(A)** Angiography at the root of the aorta. The right coronary artery could be perfused with contrast agent, but the left coronary artery was missing. **(B)** Selective right coronary artery angiography demonstrated a dilated right coronary artery. **(C)** Delay radiological exposure revealed that the left ventricle could be supplied by the right coronary artery, which was considered the right coronary artery-dependent left coronary artery circulation. **(D)** The angiography failed to illustrate the left coronary artery in the left coronary sinus. **(E)** A strange curve-like migration of the origin of the left coronary artery by angiography at a superior location. **(F)** Then, the left coronary artery could be perfused at an extremely low volume. **(G)** Anatomic aspect of the axial section of aortic and pulmonary valves and left coronary artery arising from the non-coronary sinus of Valsalva with an intramural segment course. RCA, right coronary artery; LCA, left coronary artery; R, right coronary sinus; L, left coronary sinus; N, non-coronary sinus; P, pulmonary artery.

To further validate the results obtained from CTA, transcatheter angiography was performed. Angiography at the root of the aorta showed that the right coronary artery could be perfused with contrast agent, but the left coronary artery was missing (Figure 4A). Selective right coronary artery angiography demonstrated a dilated right coronary artery (Figure 4B). Delay radiological exposure revealed that the left ventricle could be supplied by the right coronary artery, which was considered right coronary artery-dependent left coronary artery circulation (Figure 4C). Compared to the CTA images, angiography illustrated the left coronary artery originated from the non-coronary sinus (Figure 4D). However, a strange curve, such as migration of the origin of the left coronary artery, was observed by angiography at a superior location (Figure 4E). Then, the left coronary artery could be perfused as an extremely low volume (Figure 4F). Thus, we suspected that the left coronary artery originated from the non-coronary sinus with a long intramural course exits the left sinus of Valsalva, giving the impression of normal origin by echocardiography.

After that, the patient immediately received surgical correction with a modified unroofing procedure. AAOCA from the non-coronary sinus of Valsalva with an initial intramural segment was diagnosed intraoperatively as well, which matched our suspicion based on angiography. The intramural wall segment finally exited from the margin of the left-right-coronary sinus, generated a sharp angle and coursed between the aorta and the pulmonary artery with general severe stenosis, resulting in severe coronary artery dysplasia (Figure 4G). After surgery, aspirin, diuretics and angiotensin-converting enzyme inhibitors were administered for a long time. Cardiac MRI post-surgery presented a normal myocardial perfusion (Figure 3F).

### Molecular Results

To exclude any genetic mutation-induced cardiomyopathy or syndrome, whole exon sequencing (WES) was performed. A peripheral blood sample was obtained from the patient in an ethylenediaminetetraacetic acid anticoagulant blood sample tube and then stored at 4°C for less than 6 h. DNA was extracted using the Blood Genome Column Medium Extraction Kit (Tiangen Biotech, Beijing, China) in accordance with the manufacturer’s instructions. Protein-coding exome enrichment was performed using the xGen Exome Research Panel v.1.0. Whole exon sequencing was performed using the NovaSeq 6000 platform (Illumina, San Diego, CA, United States), and the raw data were processed using FastP to remove adapters and filter out low-quality reads. Paired-end reads were aligned to the Ensembl GRCh38/hg38 reference genome using the Burrows–Wheeler Aligner. Variant annotation was performed in accordance with database-sourced minor allele frequencies (MAFs) and practical guidelines on pathogenicity issued by the American College of Medical Genetics. The annotation of MAFs was performed based on the 1,000 Genomes, dbSNP, ESP, ExAC, Provean, Sift, Polypen2_hdiv, Polypen2_hvar, and Chigene in-house MAF databases using R software (R Foundation for Statistical Computing, Vienna, Austria). After retrieving all the potential variants based on WES, there was no identical genic mutation related to his clinical phenotype identified, and the patient was negative for any proven cardiomyopathy or familial inheritance of arrhythmia-associated mutations. Additionally, potential metabolic disorders were also excluded by the WES analysis.

### Outcome and Follow-Up

After 1 month of intensive care and positive treatment, the patient was discharged and remained asymptomatic for myocardial ischemia. However, cardiac function could not recover to normal levels, and follow-up echocardiography demonstrated a drop in LVEF of 40–45%. During his 18-month follow-up, the patient was engaged in slight and mild exercise only. The markers of myocardial injuries recovered gradually, and the ST segment of EKG returned to baseline level. Furthermore, myocardial fibrosis was terminated according to the cardiac MRI scanning at 1 year post operation.

## Discussion

Cardiogenic syncope is one of the most dangerous presentations, which leads to high potential onset of SCD, threatening healthy outcomes among high-risk populations. Genetic and environmental factors are identified to be involved in the events of syncope or SCD attacks. Typically, critical arrhythmia, hypertrophic cardiomyopathy, congenital heart malformation and congenital or required coronary artery disease are significantly associated with attacks of SCD (11). In an autopsy study of college athletes who suffered SCD, congenital coronary artery malformation was the top identified reason responding for this adverse event (12). Therefore, AAOCA should be considered in young patients with unexplained as the top cause.

Traditionally, congenital anatomical variation of the coronary artery can be classified as abnormalities of coronary artery origin, course, destination, and various vessels. AAOCA generally refers to a kind of congenital coronary artery malformation in which the left or right coronary artery fails to originate normally from the corresponding aortic sinus, with or without an intramural course (13). AAOCA is caused by the deviation of the arterial trunk separation in the embryonic stage and the abnormal development or incomplete development of the coronary artery (14). While most coronary anomalies are benign, among AAOCA subtypes, ALCA arising at or above the right sinus of Valsalva with an intramural course between the aorta and the pulmonary artery has been commonly considered the most fatal type in prior studies (15–17). In addition, the existing angle between the coronary ostium and proximal segment has also been emphasized as a crucial risk factor in SCDs (18). The most common pathogenic mechanisms of coronary dysfunction related to the intramural coronary course are identified as variable lateral compression and stenosis inside the aortic tunica media (19). This compression can present at the rest stage or display when performing intensive exercise. However, the formation of lateral branches that originate from the “right” coronary artery can improve compensatory circulation, rescue the ischemic regions, and relieve symptoms. Sometimes, functional compensation for a healthy coronary artery causes delayed diagnosis and surgical treatment for AAOCA. The failure to diagnose AAOCA leads to recurrent attacks of syncope, resulting in irreversible myocardial ischemia and SCD. Moreover, AAOCA can be easily misdiagnosed as fulminant myocarditis or cardiomyopathy without detailed coronary artery-based radiographic examination. Therefore, it is challenging to identify AAOCA patients early.

Currently, individuals who are suspected with AAOCA are recommended to undergo transthoracic echocardiography, non-invasive coronary CTA and cardiac MRI. The combination of several imaging strategies is reasonable to better screen the coronary artery anatomy and to achieve an accurate diagnosis in children (20). In most cases, coronary CTA can identify the location of the anomalous origin, details of the intra-arterial segment, and the angle between the coronary ostium and proximal segment (21, 22). Cardiac MRI has the advantages of providing coronary artery and functional imaging, particularly in evaluating the area and severity of myocardial infarction, and illustrating the course of coronary arteries in most patients (23). Moreover, transcatheter angiography could provide an adjunct to determine the detailed coronary artery anomalies. However, it is an invasive examination, which limits its application as a routine method, as CTA and cardiac MRI might achieve the imaging goal in most cases. In this case, the combination of coronary CTA and cardiac MRI (non-invasive protocol) failed to demonstrate the accurate origin and course of the left coronary artery due to the extreme severity of intramural stenosis and the unique coronary existing. Therefore, non-invasive examination could not identify the real reason for myocardial ischemic attack. On this occasion, more reliable measurements of intramural segments were obtained by transcatheter angiography (invasive protocol), which is the most recommended method of classifying the anatomical structure and severity of AAOCA, and benefit the strategy design for surgical treatment.

Therefore, proper selection for non-invasive and invasive protocols is critical for coronary anatomic and functional evaluation. Early recognition and intervention could reduce malignant events. The unroofing technique has been confirmed as a safe and reliable surgical method for ALCA (24, 25). However, how to treat asymptomatic patients with incidental findings of ALCA from the opposite sinus with an inter-arterial course is still controversial. Avoiding strenuous activity is recommended in all left coronary arteries originating from the inappropriate coronary sinus, and surgical revascularization should be performed as early as possible in symptomatic cases before transient dizziness and amaurosis frequently appear during exercise, as these symptoms imply an intramural or inter-arterial course between the aorta and the pulmonary artery, with or without a narrow angle between the coronary ostium and proximal segment.

## Conclusion

In summary, AAOCA, especially ALCA, presented a major potential risk of SCD. Here, we report an atypical manifestation of ALCA arising from the inappropriate sinus of Valsalva and merging into the intramural to develop a strange course and then sprouting between the aorta and the pulmonary artery. However, normal echocardiography and CTA failed to reveal malformation of the left coronary artery. Finally, angiography and observation during surgical treatment both confirmed this diagnosis. Therefore, the diversity of AAOCA might result in a relatively normal course under non-invasive radiological imaging scanning. Further angiography would be necessary for significant separation between clinical manifestations and imaging tests.

## Data Availability Statement

The datasets for this article are not publicly available due to concerns regarding participant/patient anonymity. Requests to access the datasets should be directed to the corresponding authors.

## Ethics Statement

This study was approved by the Ethics Committee of West China Second Hospital of Sichuan University (2014-034). Written informed consent to participate in this study was provided by the participants’ legal guardian/next of kin. Written informed consent was obtained from the individual(s), and minor(s)’ legal guardian/next of kin, for the publication of any potentially identifiable images or data included in this article.

## Author Contributions

YL, FH, YH, KZ, XS, ZL, and TW were the patient’s physicians. JW and JF reviewed the literature and contributed to manuscript drafting. YL and JF performed the mutation analysis. JF and YH conceptualized and designed the study, coordinated and supervised the data collection, and critically reviewed the manuscript for important intellectual content, were responsible for the revision of the manuscript for important intellectual content. All authors issued final approval for the version to be submitted.

## Conflict of Interest

The authors declare that the research was conducted in the absence of any commercial or financial relationships that could be construed as a potential conflict of interest.

## Publisher’s Note

All claims expressed in this article are solely those of the authors and do not necessarily represent those of their affiliated organizations, or those of the publisher, the editors and the reviewers. Any product that may be evaluated in this article, or claim that may be made by its manufacturer, is not guaranteed or endorsed by the publisher.
